# The flow index provides a comprehensive assessment of erectile dysfunction by combining blood flow velocity and vascular diameter

**DOI:** 10.1038/s41598-022-19364-5

**Published:** 2022-09-27

**Authors:** Wei-Lun Huang, Sheng-Yung Tung, Chi-Shin Tseng, Tzung-Dau Wang, Wen-Jeng Lee, Jyh-Horng Chen, Yann-Ron Su, Hong-Chiang Chang, Yi-Kai Chang

**Affiliations:** 1 Department of Urology, National Taiwan University Hospital and College of Medicine, No. 7, Chung-Shan South Road, Zhongzheng District, Taipei, 100 Taiwan; 2 Department of Urology, E-Da Hospital, Kaohsiung, Taiwan; 3 Cardiovascular Center and Division of Cardiology, Department of Internal Medicine, National Taiwan University Hospital and College of Medicine, Taipei City, Taiwan; 4 Department of Medical Imaging, National Taiwan University Hospital and College of Medicine, Taipei City, Taiwan; 5 Department of Nursing, I-Shou University, Kaohsiung, Taiwan

**Keywords:** Ultrasound, Sexual dysfunction, Vascular diseases, Erectile dysfunction, Sexual dysfunction

## Abstract

Dynamic duplex sonography (DUS) is not comprehensive in the evaluation of arteriogenic erectile dysfunction (ED). We introduced a new parameter, the flow index (FI), into the assessment of arteriogenic ED. A retrospective review of a prospective database was conducted. Patients undergoing DUS and pelvic computed tomography angiography for the evaluation of ED were included. The FI was calculated from peak systolic velocity (PSV) and the percentages of pelvic arterial (PLA) stenosis. Correlations between PSV, PLA stenosis, the FI, and erectile function were calculated. Eighty-three patients were included. Compared with PSV, the FI had better correlations with the erection hardness score (EHS) (r_s_ = 0.405, *P* < 0.001 for FI; r_s_ = 0.294, *P* = 0.007 for PSV). For EHS < 3, the areas under the ROC curve of FI and PSV were 0.759 and 0.700, respectively. In patients with normal DUS but EHS < 3, PLA stenosis was more severe (62.5% vs. 10.0%, *P* = 0.015), and the FI was lower (8.35 vs. 57.78, *P* = 0.006), while PSV was not different. The FI is better than PSV in the evaluation of arteriogenic ED. On the other hand, assessment of the pelvic arterial system should be included in the evaluation of ED.

## Introduction

Nearly 50% of men aged 40–70 years suffer from erectile dysfunction (ED)^1^. The aetiology of ED can be classified as psychogenic, organic, and mixed. Arterial insufficiency or stenosis, which accounts for approximately 75% of all ED cases, is the major cause in men who have metabolic disorders or cardiovascular diseases^2–8^. The cavernous artery, which is derived from the internal iliac artery and its division, including the anterior division of the internal iliac artery, internal pudendal artery, and common penile artery, affects tumescence of the corpus cavernosum and is responsible for erection^9^.

Dynamic duplex sonography (DUS), which measures the blood flow velocity of the cavernous artery, is commonly used for the evaluation of arteriogenic ED. According to current European Association of Urology (EAU) guidelines, a peak systolic blood flow (PSV) > 30 cm/s, an end-diastolic velocity (EDV) < 3 cm/s, and a resistance index (RI) > 0.8 are generally considered normal^10^. On the other hand, multislice computed tomography angiography (CTA), which has become another diagnostic tool for arteriogenic ED, is able to clearly depict the anatomy of the pelvic arterial (PLA) system and accurately measure anatomical stenosis^11–14^.

Although current measurements from DUS and CTA are commonly used for the evaluation of arteriogenic ED, these measurements may not be comprehensive enough in theory because they provide only partial haemodynamic data, such as “velocity” or “diameter”. In fact, several studies have indicated that a diagnosis of arteriogenic ED cannot be made with to the use of PSV alone^15,16^. Furthermore, in our experience, we noticed that the results from DUS may not be comparable to clinical observations. Some patients who have normal DUS results still have poor penile hardness after intracavernous injection of prostaglandin E1 (PGE1), suggesting that current measurements from DUS may not be comprehensive enough for diagnosing vasculogenic ED. Some patients were diagnosed with pelvic arterial stenosis on subsequent CTA examination. It is possible that those patients might have insufficient penile perfusion from the narrowed vascular lumen, but the PSV might still be within the normal range because the narrowed vascular lumen might result in higher blood pressure and subsequently lead to a falsely higher blood flow velocity. For such scenarios, we created a new parameter in which the blood flow velocity and diameter of the vascular lumen are considered together, which may help to more comprehensively evaluate arteriogenic ED.

## Results

### Demographics and patient characteristics

A total of 91 patients were enrolled, of whom 8 patients who had veno-occlusive dysfunction were excluded, and 83 patients were included for final analysis. The characteristics and demographics are shown in Table 1. The median age was 61 years, and the median PSV, EDV, RI, PLA stenosis, and flow index (FI) were 36.70 cm/s, 2.56 cm/s, 0.89, 37.50%, and 28.94, respectively. Nineteen patients had a PSV < 30 cm/s. According to the simplified International Index of Erectile Function (IIEF-5) score, the numbers of patients with severe, moderate, mild to moderate, mild, and no ED were 46 (55.42%), 20 (24.10%), 14 (16.87%), 3 (3.61%), and 0 (0.00%), respectively; the numbers of patients with erection hardness scores (EHS) of 1, 2, 3, and 4 were 35 (42.17%), 27 (32.53%), 19 (22.89%), and 2 (2.41%), respectively. More than half of the enrolled patients had IIEF-5 score < 8 or EHS < 3. Notably, of the 34 patients who had normal DUS (PSV > 30 cm/s, EDV < 3 cm/s, and RI > 0.8), 25 patients (73.53%) had EHS < 3.

**Table 1:** Characteristics of the enrolled population.

Continuous variables	Whole cohort (n = 91)		Patients without venous leak (n = 83)	
	Median	(Range)	Median	(Range)
Age (year)	61	(37–78)	61	(37–78)
BMI (kg/m^2^)	26.00	(15.57–38.51)	25.88	(15.57–38.51)
IIEF-5 score	5	(0–19)	5	(0–19)
EHS	2	(1–4)	2	(1–4)
PLA stenosis (%)	40.00%	(0.00–100.00%)	37.50%	(0.00–100.00%)
PSV (cm/s)	36.80	(7.21–91.2)	36.70	(7.21–91.2)
EDV (cm/s)	3.21	(− 6.22 to 9.88)	2.56	(− 6.22 to 9.88)
RI	0.89	(0.57–1.15)	0.89	(0.57–1.15)
FI	26.43	(0.00–106.54)	28.94	(0.00–106.54)

Categorical variables	Whole cohort (n = 91)		Patients without venous leak (n = 83)	
	Number	(%)	Number	(%)
Hypertension	52	(57.14)	46	(55.42)
CAD	34	(37.36)	29	(34.94)
PAOD	8	(8.79)	7	(8.43)
Diabetes mellitus	35	(38.46)	30	(36.14)
Hyperlipidaemia	37	(40.66)	34	(40.96)
Smoking habit	19	(20.88)	15	(18.07)
Alcohol use	9	(9.89)	7	(8.43)
Prostate enlargement	28	(30.77)	28	(33.73)
IIEF-5 score < 8	52	(57.14)	46	(55.42)
IIEF-5 score 8–11	20	(21.98)	20	(24.10)
IIEF-5 score 12–16	16	(17.58)	14	(16.87)
IIEF-5 score 17–21	3	(3.30)	3	(3.61)
IIEF-5 score > 21	0	(0.00)	0	(0.00)
EHS 1	40	(43.96)	35	(42.17)
EHS 2	27	(29.67)	27	(32.53)
EHS 3	22	(24.18)	19	(22.89)
EHS 4	2	(2.20)	2	(2.41)

Median PSV and RI were within normal reference, while more than half of patients had IIEF < 8 or EHS < 3.

*BMI* body mass index, *CAD* coronary artery disease, *EDV* end-diastolic velocity, *EHS* erection hardness score, *FI* flow index, *IIEF-5* the simplified International Index of Erectile Function, *PAOD* peripheral arterial occlusive disease, *PLA* pelvic artery, *PSV* peak systolic velocity, *RI* resistance index.

### Correlation with erectile function and predictive value of the FI

**Figure 1:**
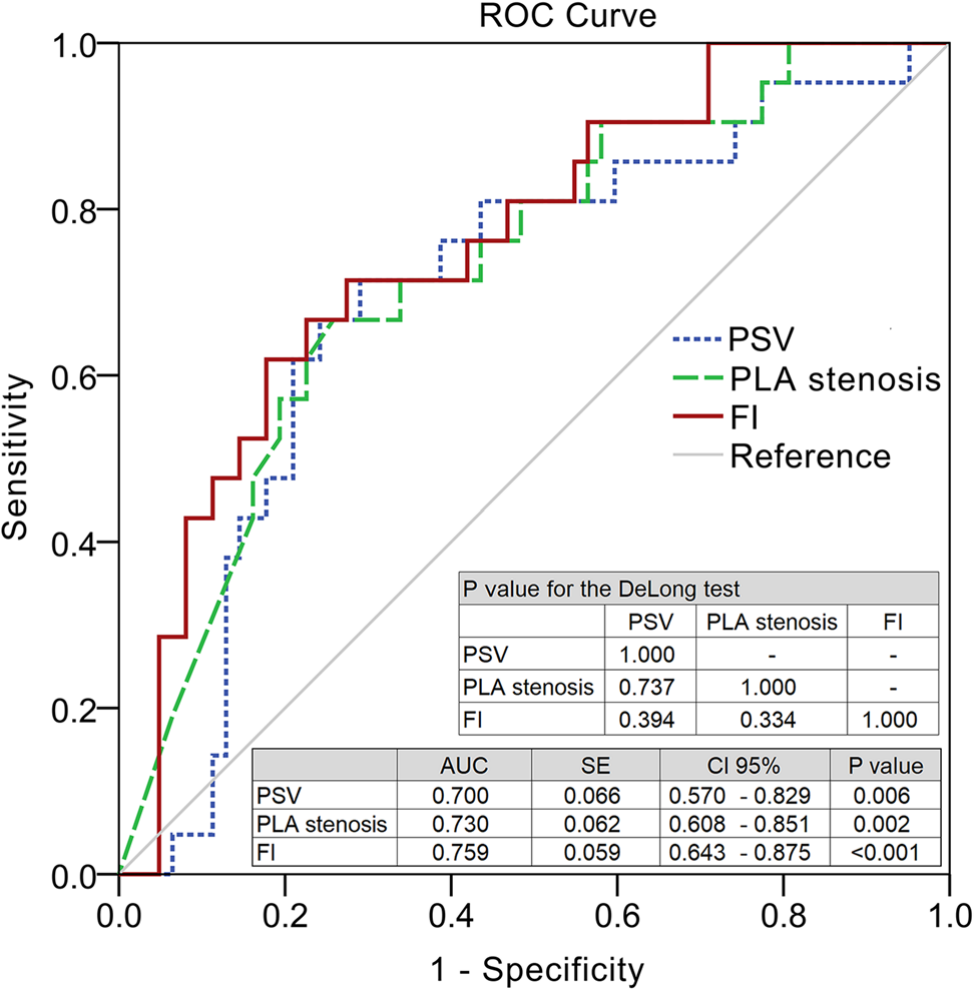
ROC Curve of EHS < 3. PSV, PLA stenosis, and FI exhibited acceptable AUCs in ROC analysis. AUC = 0.5 (no discrimination), 0.5–0.6 (poor discrimination), 0.6–0.7 (fair discrimination), 0.7–0.8 (acceptable discrimination), 0.8–0.9 (excellent discrimination), and 0.9–1.0 (outstanding discrimination). The DeLong test of AUC revealed no significant difference between the AUCs of PSV, PLA stenosis, and FI. EHS: erection hardness score, FI: flow index, PSV: peak systolic velocity, PLA: pelvic artery.

Spearman’s rank correlation analysis is shown in Table 2. PSV had a weak correlation with the EHS (r_s_ = 0.294, *P* = 0.007); PLA stenosis had weak correlations with the EHS and IIEF-5 score (r_s_ = − 0.355, *P* < 0.001 for EHS; r_s_ = -0.253, *P* = 0.021 for IIEF-5 score); EHS had a moderate correlation with IIEF-5 score (r_s_ = 0.546, *P* < 0.001). However, compared with both PSV and PLA stenosis, the FI had the best correlation with the EHS and IIEF-5 score (r_s_ = 0.405, *P* < 0.001 for EHS; r_s_ = 0.323, *P* = 0.003 for IIEF-5 score). PSV and PLA stenosis had a moderate and a very strong correlations with the FI (r_s_ = 0.513, *P* < 0.001 for PSV; r_s_ = − 0.919, *P* < 0.001 for PLA stenosis). In the receiver operating characteristic (ROC) analysis, PSV, PLA stenosis, and FI had acceptable areas under the ROC curve (AUCs) for an EHS < 3 (AUC = 0.700 for PSV; AUC = 0.730 for PLA stenosis; AUC = 0.759 for FI). The AUCs of the three parameters were compared using the DeLong test (*P* = 0.737 for PSV and PLA stenosis; *P* = 0.394 for PSV and FI; *P* = 0.334 for PLA stenosis and FI) (Fig. 1). The cut-off values identified by the Youden index were 33.97 cm/s, 21.50%, and 52.47 for the PSV, PLA stenosis, and FI, respectively.

**Table 2:** Correlation between erectile function and objective measurements.

Parameters		EHS	IIEF-5	PSV	PLA stenosis	FI
EHS	r_s_	1.000				
	*P* value					
IIEF-5	r_s_	0.546*	1.000			
	*P* value	< 0.001				
PSV	r_s_	0.294*	0.206	1.000		
	*P* value	0.007	0.062			
PLA stenosis	r_s_	− 0.355*	− 0.253*	− 0.248*	1.000	
	*P* value	< 0.001	0.021	0.024		
FI	r_s_	0.405*	0.323*	0.513*	− 0.919*	1.000
	*P* value	< 0.001	0.003	< 0.001	< 0.001	

The FI had better correlations with erectile function than PSV.

*EHS* erection hardness score, *FI* flow index, *IIEF-5* the simplified International Index of Erectile Function, *PLA* pelvic artery, *PSV* peak systolic velocity.

Spearman's rank correlation coefficient, *P < 0.05.

### Patients with EHS < 3 who had normal DUS

Patients with EHS < 3 who had normal DUS (Group 1) had similar PSV but a lower FI and more severe PLA stenosis than patients who had EHS 3–4 (Group 2) (median: 31.35 cm/s vs. 36.60 cm/s, P = 0.115 for PSV; 8.36 vs. 57.78, *P* = 0.006 for FI_;_ 62.50% vs. 10.00%, *P* = 0.015 for PLA stenosis) (Table 3). By using the cut-off values identified by the ROC analysis, 18 patients in group 1 (72.0%) had an FI lower than the cut-off value, while only 8 patients in group 2 (38.0%) had an FI lower than the cut-off value (*P* value = 0.021, chi-square test).

**Table 3:** Difference between patients with normal DUS but EHS < 3 (group 1) and patients with EHS 3–4 regardless of DUS results (group 2).

Parameters	Groups	Number	Median	(Range)	*P* value
PSV (cm/s)	Group 1	25	31.35	(56.65–22.35)	0.115
	Group 2	21	36.60	(53.60–15.00)	
FI	Group 1	25	8.36	(104.30–0.00)	0.006*
	Group 2	21	57.78	(88.70–1.66)	
PLA stenosis (%)	Group 1	25	62.50%	(100.00%–0.00)	0.015*
	Group 2	21	10.00%	(85.00%–0.00)	

Compared with patients who had EHS 3–4, patients who had normal DUS but poor EHS had a significantly lower FI and more severe PLA stenosis.

*EHS* erection hardness score, *FI* flow index, *IIEF-5* the simplified International Index of Erectile Function, *PLA* pelvic artery, *PSV* peak systolic velocity.

Mann–Whitney U Test, *P < 0.05.

### Patients with different underlying diseases

The effects of the different underlying diseases on erectile function, PSV, PLA stenosis, and FI are shown in Supplementary Table S1. EHS was significantly lower in patients with diabetes mellitus (DM) (median: 1.0 vs. 2.0, *P* = 0.016), while IIEF-5 scores were nearly significantly lower in patients with hypertension (HTN), DM, and smoking habits (median: 5 vs. 9, *P* = 0.058 for HTN; 5 vs. 8.5, *P* = 0.095 for DM; 4 vs. 5.5, *P* = 0.058 for smoking habits). The FI was significantly lower in patients with HTN, DM, and hyperlipidemia (HLP) and nearly significantly lower in patients with smoking habits (median: 23.24 vs. 42.56, *P* = 0.021 for HTN; 2.66 vs. 42.29, *P* < 0.001 for DM; 14.43 vs. 38.46 *P* = 0.027 for HLP; 2.66 vs. 32.30, *P* = 0.076 for smoking habits). PLA stenosis was more severe in patients with HTN, DM, and HLP (median: 47.50% vs. 25.00%, *P* = 0.036 for HTN; 81.25% vs. 21.50%, *P* = 0.001 for DM; 53.13% vs. 30.00%, *P* = 0.088 for HLP). However, PSV was not significantly different in these patients.

## Discussion

To the best of our knowledge, this is the first study to combine both penile Doppler and angiography to evaluate erectile dysfunction. Our study demonstrated that the FI had a better correlation with erectile function and might be more predictive than PSV and arterial stenosis for arteriogenic ED. Smoking and metabolic diseases, such as HTN, DM, and HLP, were associated with poor erectile function and low FI, with which PSV was not significantly associated. Furthermore, patients who had poor penile perfusion results might still have normal PSV, while the FI was significantly lower in those patients. Therefore, the combination of PSV and arterial stenosis is more valuable and comprehensive in the assessment of penile blood flow.

The utility of DUS in the evaluation of penile arterial insufficiency has been widely discussed, and cut-off values of PSV for diagnosing arteriogenic ED range from 25 to 35 cm/s in different studies^17–21^. According to the current consensus, PSV > 30 cm/s is considered normal, while PSV < 25 cm/s is diagnostic of arterial insufficiency. PSV between 25 and 30 cm/s is intermittent and may be considered partial arterial insufficiency. On the other hand, EDV < 3 cm/s or RI > 0.8 are exclusive for venous leakage, while EDV > 6 cm/s or RI < 0.6 are accepted as an indicator of venous leakage. The penile vascular status can be determined with the result from DUS and clinical presentation^10^. However, in our study, approximately 70% of patients who had normal DUS results still had a poor EHS after intracavernous injection of PGE1. Other studies have also demonstrated that PSV had a low AUC for diagnosing arteriogenic ED, while other measurements, such as age-matched PSV and acceleration time, were more accurate^15,16^. In fact, cut-off values of DUS parameters from different studies are widely variable, which may indicate the existence of undiscovered measurements other than Doppler parameters. Thus, more precise measurements are required for accurate evaluation of penile vascular status.

In addition to DUS, the diameter of the artery is another assessment for anatomical patency. However, evaluating the patency of fine vessels was difficult with noninvasive tools, and thus, percutaneous transluminal angiography (PTA) used to be the main tool for the assessment^10^. Despite its high accuracy, PTA is invasive and expensive and requires hospitalization and careful postprocedure care. Fortunately, since the development of multislice CTA and three-dimensional imaging techniques, arterial patency can be evaluated noninvasively. Research has demonstrated that multislice coronary CTA possesses high diagnostic accuracy for the detection of obstructive coronary stenosis and is an effective alternative to invasive PTA^22^. Recently, several studies have reported the feasibility of multislice CTA in the evaluation of penile arterial patency and vasculogenic ED^11,12,23,24^. By using PTA as the standard tool, the result of arterial stenosis obtained from multislice CTA was comparable to that from PTA, with a sensitivity of 96%, a specificity of 43%, and an accuracy of 79%^11^. Moreover, the PERFECT-1 study revealed that CTA could provide clear information on the whole pelvic arterial system and characteristics of stenosis and could be further used as a guide for pelvic endovascular therapies^23^. In our study, we successfully demonstrated the utility of multislice CTA and the coordination of multislice CTA and DUS. Thus, multislice CTA should be considered an effective tool for the evaluation of penile arterial patency performed before invasive PTA.

Reportedly, smoking and metabolic syndrome negatively affect vascular health and erectile function^25,26^. Our results indicate that FI could detect a decrease in penile blood perfusion in patients who smoke or have metabolic syndrome. The reason why PSV was not predictive in the analysis is unclear. We speculated that the narrowed lumen of unhealthy vessels might result in higher blood pressure and lead to higher blood flow velocity. Therefore, FI may be more valuable in the evaluation of ED in these patients.

By combining PSV and PLA patency, the FI has better predictive value for arteriogenic ED and can explain why some patients, for whom arterial stenosis may be the main cause, have normal DUS data but still have a poor EHS after intracavernous PGE1 injection. Therefore, even though PSV may be useful in the evaluation of arteriogenic ED in general, accuracy will be better if arterial stenosis is also taken into account. The cut-off value of the FI may not be used for the initial diagnosis of ED because patients who have clinical ED and arterial stenosis will still be regarded as having arteriogenic ED despite a normal FI value; however, our results showed that the FI is more valuable for describing penile arterial perfusion and can be used as a parameter for assessing treatment outcome.

Patients with arteriogenic ED still respond to phosphodiesterase type 5 (PDE5) inhibitors^27–29^. In patients with mild-to-moderate ED, up to 60% had normal erectile function after treatment with sildenafil 50 mg for 1 year^27^. The response was significantly better in patients receiving nightly treatment than in those receiving as-needed treatment. The treatment effect could persist in patients with nightly treatment after a 6 months off-treatment period. These effects were similar in an animal model^30,31^. These results indicate that the long-term use of PDE5 inhibitors might restore normal erectile tissue by improving endothelial function and penile perfusion. However, one study reported that patients with arteriogenic ED seemed to respond less to PDE5 inhibitors than patients with venous insufficiency or non-arteriogenic ED^29^. The results indicate that an accurate diagnosis of arteriogenic ED is crucial for the treatment strategy. Theoretically, FI would increase after treatment with the PDE5 inhibitor; however, we did not analyse the effect of PDE5 inhibitors on FI in our study; therefore, further study is required.

The study has several limitations. First, men with normal erectile function were not included in our study. Our results were mainly based on the population with subjective and/or objective ED, and thus, the actual cut-off value of the FI might be different in the general population. Second, the kinds of premedication before DUS and CTA were different. Intracavernosal injection of alprostadil was administered prior to DUS, while sublingual nitroglycerine was given before CTA. Effects on penile haemodynamics and vasodilation might be different between the two kinds of premedication, which might lead to potential biases. Third, accurate measurement of the diameter of the cavernous artery was difficult by using DUS because the diameter varies between different segments even though the target was always the proximal penis. Thus, we chose CTA to depict arterial patency and measure arterial stenosis.

On the other hand, the strengths of our study are noteworthy. First, although the study was retrospective, the data were collected from a prospective database. All surveys were administered in a prospective attempt. CTA and DUS were interpreted individually by different specialists. The specialists interpreting CTA did not know the result of DUS, nor did the specialist performing DUS know the result of CTA. Second, we used objective EHS after intracavernous injection with PGE1 during DUS as the parameter, in which erectile function could be evaluated in real time together with the haemodynamic profile and objective hardness of the penis rather than self-reported questionnaires. Recall bias from the patients could also be minimized. Most importantly, this is the first study that introduces the concept of volumetric flow rate into the evaluation of penile arterial perfusion. Although study for further clinical utility is required, FI is theoretically more precise than using DUS or CTA alone. The current study not only provides a better measurement but also brings a new perspective to medical specialists.

## Conclusion

Assessment of penile perfusion can be more comprehensive if both blood flow velocity and arterial stenosis are considered together. By using DUS and CTA, our results not only demonstrate the feasibility of using the FI to evaluate penile perfusion but also show that the FI is a more accurate assessment than PSV for evaluating arteriogenic ED.

## Methods

### Ethics approval

The present study followed all ethical standards concerning experimentation and research. The Institutional Review Board of National Taiwan University Hospital approved our study (Approval Number: 201804044RINA). All patients provided written informed consent approved by the institutional ethics committee. All experiments were performed in accordance with relevant guidelines and regulations.

### Study design and patient enrolment

We conducted a retrospective review from a prospective, unblinded, and single-arm database. From September 2008 to August 2017, patients who visited our medical centre for ED and subsequently underwent DUS and CTA were enrolled. The interval between CTA and DUS should be less than 6 months. We excluded patients who had (1) penile veno-occlusive dysfunction, characterized by both PSV > 30 cm/s and RI < 0.8, (2) arterial inflow to the penis entirely from the accessory pudendal arteries rather than the usual internal pudendal artery and common penile artery, (3) previous pelvic angiography or any pelvic vascular intervention, (4) previous radical prostatectomy or pelvic radiation, (5) Peyronie’s disease, (6) untreated hypogonadism (serum total testosterone < 3 mg/L within 14 days before enrolment), (7) acute coronary syndrome, stroke, or life-threatening arrhythmia within 3 months before enrolment, (8) poorly controlled diabetes mellitus with HbA1C levels > 9%, (9) serum creatinine levels > 2.5 mg/dL, (10) bleeding tendency or hypercoagulopathy, (11) life expectancy < 12 months, and (12) known intolerance to contrast agents. According to the current consensus, ED is defined either as (1) the inability to attain and/or maintain penile erection sufficient for satisfactory sexual performance or (2) the consistent or recurrent inability to attain and/or maintain penile erection sufficient for sexual satisfaction^32,33^. The severity of ED was based on the IIEF-5 obtained at the interview before any procedure, which was defined as severe (5–7), moderate (8–11), mild to moderate (12–16), mild (17–21), and no ED (22–25)^34^. Demographics were obtained from medical records.

### Dynamic duplex sonography

**Figure 2:**
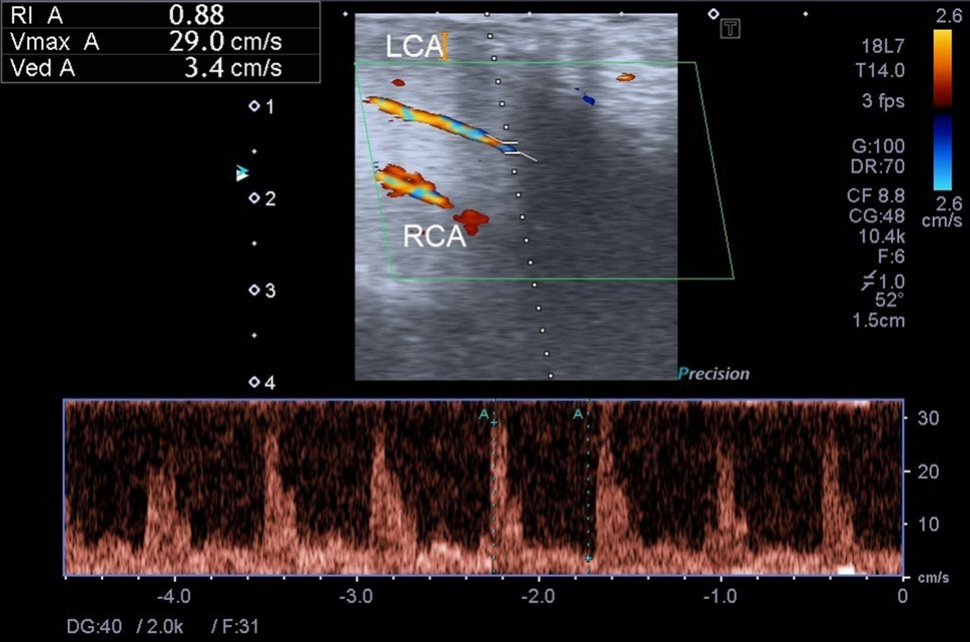
An example of dynamic duplex sonography of the penis. Both LCA and RCA are shown in this sonography. The hemodynamic parameters of the LCA are measured. The PSV (shown as Vmax A) is 29.0 cm/s, the EDV (shown as Ved A) is 3.4 cm/s, and the RI (shown as RI A) is 0.88. EDV: end-diastolic velocity, LCA: left cavernous artery, PSV: peak systolic velocity, RCA: right cavernous artery, RI: resistance index.

DUS was performed with a high-resolution solid-state linear array ultrasound transducer with a real-time image colour Doppler ultrasound scanner (7.5–12 MHz frequency) by an experienced andrologist (Fig. 2). An intracavernous injection of alprostadil (20 μg) was administered 10 min before the DUS exam, and the ultrasound transducer was placed over the penile root for continuous measurement of the cavernous artery^3,35^. The EHS was objectively observed by the andrologist during the artificial erection. EHS 1 indicated that the penis was enlarged but not hard; EHS 2 indicated that the penis was hard but not hard enough for penetration; EHS 3 indicated that the penis was hard enough for penetration but not completely hard; EHS 4 indicated that the penis was completely hard and fully rigid^36^. In this study, EHS < 3 was defined as the cut-off value in the ROC analysis. A PSV > 30 cm/s, an EDV < 3 cm/s, and an RI > 0.8 were considered normal^10,37^. Because the penis was supplied by the bilateral cavernous arteries, PSV of both sides was considered in the analyses. Therefore, we used the mean PSV of the bilateral cavernous arteries for the analyses rather than the higher or lower PSV measured from the right or left arteries. Correlation and ROC analyses from the lower PSV, higher PSV, and mean PSV of both sides were performed (Supplementary Table S2 and Supplementary Fig. S1).

### Pelvic computed tomography angiography

The application of pelvic CTA in penile vascular evaluation has been well demonstrated in our hospital^12,13,23^. This procedure was performed with a multislice 64-detector row CT scanner (LightSpeed VCT; GE Healthcare, Milwaukee, WI, USA), with all patients receiving 0.6 mg sublingual nitroglycerine as premedication. Images were reconstructed with a 0.625 mm slice thickness and 22 cm field of view. Three-dimensional reconstruction was applied. In accordance with Wang et al.^38^, the whole PLA system from the common iliac artery to the distal penile artery was assessed and classified into 8 zones (Zone 1: common iliac artery, Zone 2: internal iliac artery, Zone 3: anterior division of the internal iliac artery, Zone 4A: proximal internal pudendal artery, Zone 4B: distal internal pudendal artery, Zone 5A: common penile artery, and Zone 5B: distal penile artery, including the cavernous artery and dorsal penile artery). All images were interpreted independently by a professor of cardiology and a professor of radiology with 10 years of experience in interpreting cardiovascular CT. A third observer was called in if there was a discrepancy in interpretation, and a consensus was reached by all three.

The stenotic severity of each segment was defined as the percentage of the reference vessel diameter at the narrowest site, according to the worst angiographic view. To properly include the effects of all segments, we defined ipsilateral PLA stenosis as follows:

$$S_{PLA} = 100\% - (100\% - S_{Z1} )(100\% - S_{Z2} ) \cdots \left( {100\% - S_{Z5B} } \right)$$

where $S_{PLA}$ is the PLA stenosis, and $S_{Zn}$ is the stenotic percentage of the referred zone of PLA system.

Both the left and right PLA stenoses were calculated, and the mean of bilateral PLA stenosis was used in the analysis.

### Measurement of the flow index

**Figure 3:**
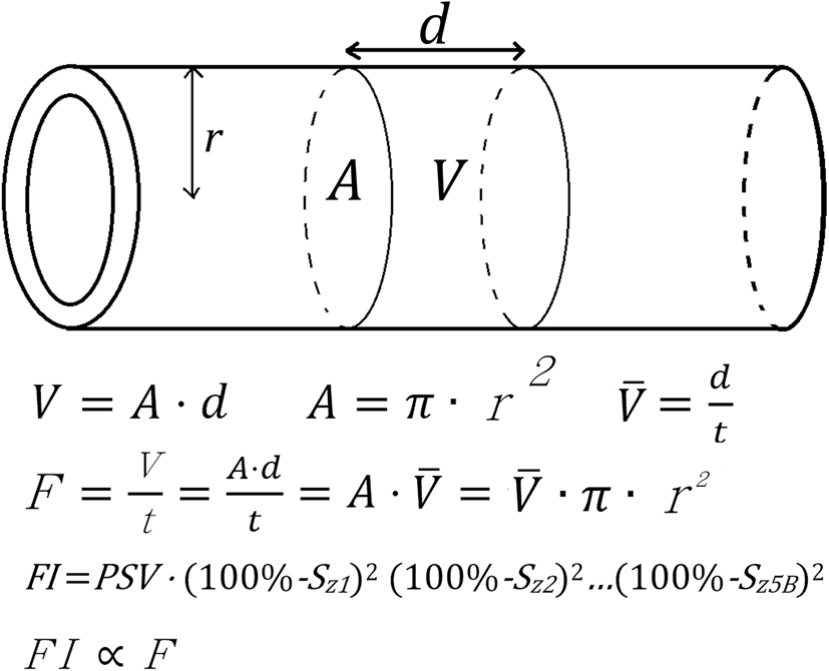
Derivation of volumetric flow rate and the FI. Volumetric flow rate is the volume infused per unit of time; volume is the product of area multiplied by distance. The formula can be expressed as $F = \frac{V}{t} = \frac{A \cdot d}{t}$, where $A$ is the area, $d$ is the distance, *F* is the volumetric flow rate, $t$ is the time, and *V* is the volume. Area is the product of π$\cdot$ radius^2^; velocity is the quotient of distance divided by the time. The formula can be further expressed as $F = \frac{V}{t} = \frac{A \cdot d}{t} = A \cdot \overline{V} = \overline{V} \cdot \pi \cdot r^{2}$, where π is the circular constant, $r$ is the radius, and $\overline{V}$ is the velocity. The radius can be expressed as 100%-stenosis% measured by CTA; velocity can be expressed as PSV measured by DUS. Thus, we used the FI as to replace volumetric flow rate in measuring arterial perfusion. $FI = PSV \cdot \left( {100\% - S_{Z1} } \right)^{2} \left( {100\% - S_{Z2} } \right)^{2} \cdots \left( {100\% - S_{Z5B} } \right)^{2}$, where $S_{zn}$ is the stenotic percentage of the referred zone of PLA system. CTA: computed tomography angiography, DUS: dynamic duplex sonography, FI: flow index, PSV: peak systolic velocity.

We introduced the concept of volumetric flow rate into our study. The volumetric flow rate was the volume of fluid movement per unit of time, which could be estimated by the following equation (Fig. 3):

$$F = \frac{V}{t} = \overline{V} \cdot \pi r^{2}$$

Where *F* is the volumetric flow rate, *V* is the volume of fluid movement, *t* is the time, $\overline{V}$ is the fluid velocity, $\pi$ is the circular constant, and $r$ is the radius.

Subsequently, the relationship among the volumetric flow rate, velocity and the radius can be expressed as:

$$F \propto \overline{V} \cdot r^{2}$$

By using PSV and vascular diameter, the volumetric flow rate of the cavernous artery could be calculated. However, the diameters of the cavernous artery varied between different segments, in which measurement error might severely interfere with the study results. On the other hand, pelvic angiography has been well demonstrated in our hospital. Therefore, we used the percentage of arterial stenosis obtained from CTA. Because the volumetric flow rate was proportional to the square of radius, we further defined a new parameter, the flow index (FI), expressed as:

$$FI = PSV \cdot \left( {100\% - S_{Z1} } \right)^{2} \left( {100\% - S_{Z2} } \right)^{2} \cdots \left( {100\% - S_{Z5B} } \right)^{2}$$

where *FI* is the flow index, *PSV* is the peak systolic velocity expressed in centimetres per second (cm/s), and *S*_*Zn*_ is the stenotic percentage of the referred zone of PLA system. The FI was defined as a score that had no physical unit because the FI was not a real physical quantity but a calculated parameter based on the concept of volumetric flow rate.

By using PSV of the cavernous artery on one side and ipsilateral PLA stenosis, the one-sided FI could be calculated. The left side FI and right side FI were added for analysis.

### Statistical methods

All analyses were performed with SPSS (version 22; SPSS, Chicago, IL, USA). The chi-square test was used for categorical data. The Spearman’s rank correlation coefficient (r_s_) was used to evaluate the dependences between nonnormally distributed variables, which could be interpreted as a negligible correlation (0.00 ≤ r_s_ < 0.10), a weak correlation (0.10 ≤ r_s_ < 0.40), a moderate correlation (0.40 ≤ r_s_ < 0.70), a strong correlation (0.70 ≤ r_s_ < 0.90), or a very strong correlation (0.90 ≤ r_s_ ≤ 0.10)^39^. Mean values were compared with an independent-samples t test, and median values were compared with the Mann–Whitney U test. ROC curves and AUCs were used to assess the diagnostic values of different parameters. The DeLong test was used to exam the differences of AUCs. Cut-off values were identified based on the Youden index. In all cases, two-tailed *P* < 0.05 was considered indicative of statistical significance.

## Supplementary Information

Supplementary Information.

## Data Availability

The datasets generated and/or analysed during the current study are available from the corresponding author on reasonable request.
