# Supplementary material for: The flow index provides a comprehensive assessment of erectile dysfunction by combining blood flow velocity and vascular diameter
Source: Sci Rep. 2022 Sep 27;12:16099. doi: 10.1038/s41598-022-19364-5 (PMC9515177; doi:10.1038/s41598-022-19364-5)
Supplement: Supplementary file 1 — Supplementary Information. [file 41598_2022_19364_MOESM1_ESM.pdf]

Supplementary Information

Manuscript Title: The Flow Index Provides a Comprehensive Assessment of Erectile Dysfunction by Combining Blood Flow Velocity and Vascular Diameter

Authors: Wei-Lun Huang[1,2], Sheng-Yung Tung[1], Chi-Shin Tseng[1], Tzung-Dau Wang[3], Wen-Jeng Lee[4], Jyh-Horng Chen[1], Yann-Ron Su[1], Hong-Chiang Chang[1], Yi-Kai Chang[1*]

[1]Department of Urology, National Taiwan University Hospital and College of Medicine, Taipei, Taiwan

[2]Division of Urology, Department of Surgery, E-Da Hospital, Kaohsiung, Taiwan

[3]Cardiovascular Center and Division of Cardiology, Department of Internal Medicine, National Taiwan University Hospital and College of Medicine, Taipei City, Taiwan

[4]Department of Medical Imaging, National Taiwan University Hospital and College of Medicine, Taipei City, Taiwan

Supplementary Table S1.

	PSV (cm/s)		PLA stenosis (%)		FI		EHS		IIEF-5 score	
	Median	(Range)	Median	(Range)	Median	(Range)	Median	(Range)	Median	(Range)
**Hypertension**										
Yes	30.50	(5.93-56.65)	47.50%	(0.00%-100.00%)	23.24	(0.00-104.30)	2	(1-4)	5	(0-16)
No	30.80	(19.50-71.90)	25.00%	(0.00%-100.00%)	42.56	(0.00-106.54)	2	(1-4)	9	(0-19)
P value	0.150		0.036*		0.021*		0.270		0.058	
**CAD**										
Yes	30.95	(5.93-56.65)	43.46%	(0.00%-100.00%)	26.03	(0.00-88.70)	1	(1-4)	5	(0-19)
No	30.58	13.85-71.90	33.75%	(0.00%-100.00%)	30.23	(0.00-106.54)	2	(1-3)	5.5	(0-19)
P value	0.532		0.320		0.498		0.172		0.327	
**PAOD**										
Yes	32.70	(12.65-50.25)	50.00%	(5.00%-92.00%)	23.57	(0.30-38.22)	1	(1-2)	5	(0-10)
No	30.65	(5.93-71.90)	36.20%	(0.00%-100.00%)	29.28	(0.00-106.54)	2	(1-4)	5.5	(0-19)
P value	0.694		0.577		0.334		0.190		0.105	
**Diabetes mellitus**										
Yes	28.80	(12.65-51.35)	81.25%	(0.00%-100.00%)	2.66	(0.00-68.70)	1	(1-3)	5	(0-18)
No	31.78	(5.93-71.90)	21.50%	(0.00%-100.00%)	42.29	(0.00-106.54)	2	(1-4)	8.5	(0-19)
P value	0.111		0.001*		<0.001*		0.016*		0.095	
**Hyperlipidemia**										
Yes	30.30	(5.93-50.25)	53.13%	(0.00%-100.00%)	14.43	(0.00-88.70)	2	(1-4)	8	(0-19)
No	30.95	(17.50-71.90)	30.00%	(0.00%-100.00%)	38.46	(0.00-106.54)	2	(1-4)	5	(0-19)
P value	0.125		0.088		0.027*		0.604		0.557	
**Smoking habit**										
Yes	29.50	(17.50-45.05)	83.22%	(5.00%-100.00%)	2.66	(0.00-65.20)	1	(1-3)	4	(0-11)
No	31.15	(5.93-71.90)	36.20%	(0.00%-100.00%)	32.30	(0.00-106.54)	2	(1-4)	5.5	(0-19)
P value	0.249		0.255		0.076		0.350		0.058	
**Alcoholism**										
Yes	23.05	(16.90-30.20)	27.50%	(0.00%-89.00%)	26.03	(0.94-52.43)	2	(1-2)	5	(4-11)
No	31.78	(5.93-71.90)	39.88%	(0.00%-100.00%)	28.99	(0.00-106.54)	2	(1-4)	5	(0-19)
P value	0.005*		0.460		0.731		0.442		0.662	
**Prostate enlargement**										
Yes	32.7	(5.93-62.20)	47.75%	(0.00%-100.00%)	26.13	(0.00-104.30)	2	(1-4)	5	(0-16)
No	30.65	(12.65-71.90)	33.75%	(0.00%-100.00%)	29.28	(0.00-106.54)	2	(1-4)	5	(0-19)
P value	0.563		0.371		0.819		0.955		0.810	

Effects of different underlying diseases on erectile function and objective parameters.

[Medians and ranges of parameters are shown. CAD: coronary artery disease, EHS: erection hardness score, FI: flow index, IIEF-5: the simplified International Index of Erectile Function, PAOD: peripheral arterial occlusive disease, PLA: pelvic artery, PSV: peak systolic velocity, Mann–Whitney U Test, * P< 0.05]

Supplementary Table S2. Correlation between different PSVs and erectile function

		Higher PSV	Lower PSV	Mean PSV	IIEF-5 score	EHS
**Higher PSV**	rs	1.000				
	P value					
**Lower PSV**	rs	0.589*	1.000			
	P value	<0.001				
**Mean PSV**	rs	0.912*	0.847*	1.000		
	P value	<0.001	<0.001			
**IIEF-5 score**	rs	0.219*	0.137	0.206	1.000	
	P value	0.047	0.215	0.062		
**EHS**	rs	0.244*	0.261*	0.294*	0.546*	1.000
	P value	0.026	0.017	0.007	<0.001	

[Since PSV was measured from bilateral cavernous arteries, there would be a higher PSV and a lower PSV measured from the right or left arteries. The mean PSV of bilateral cavernous arteries had a better correlation with EHS, compared with the higher and the lower PSV. EHS: erection hardness score, IIEF-5: the simplified International Index of Erectile Function, PSV: peak systolic velocity. Spearman's rank correlation coefficient, * P< 0.05.]

Supplementary Fig. S1.

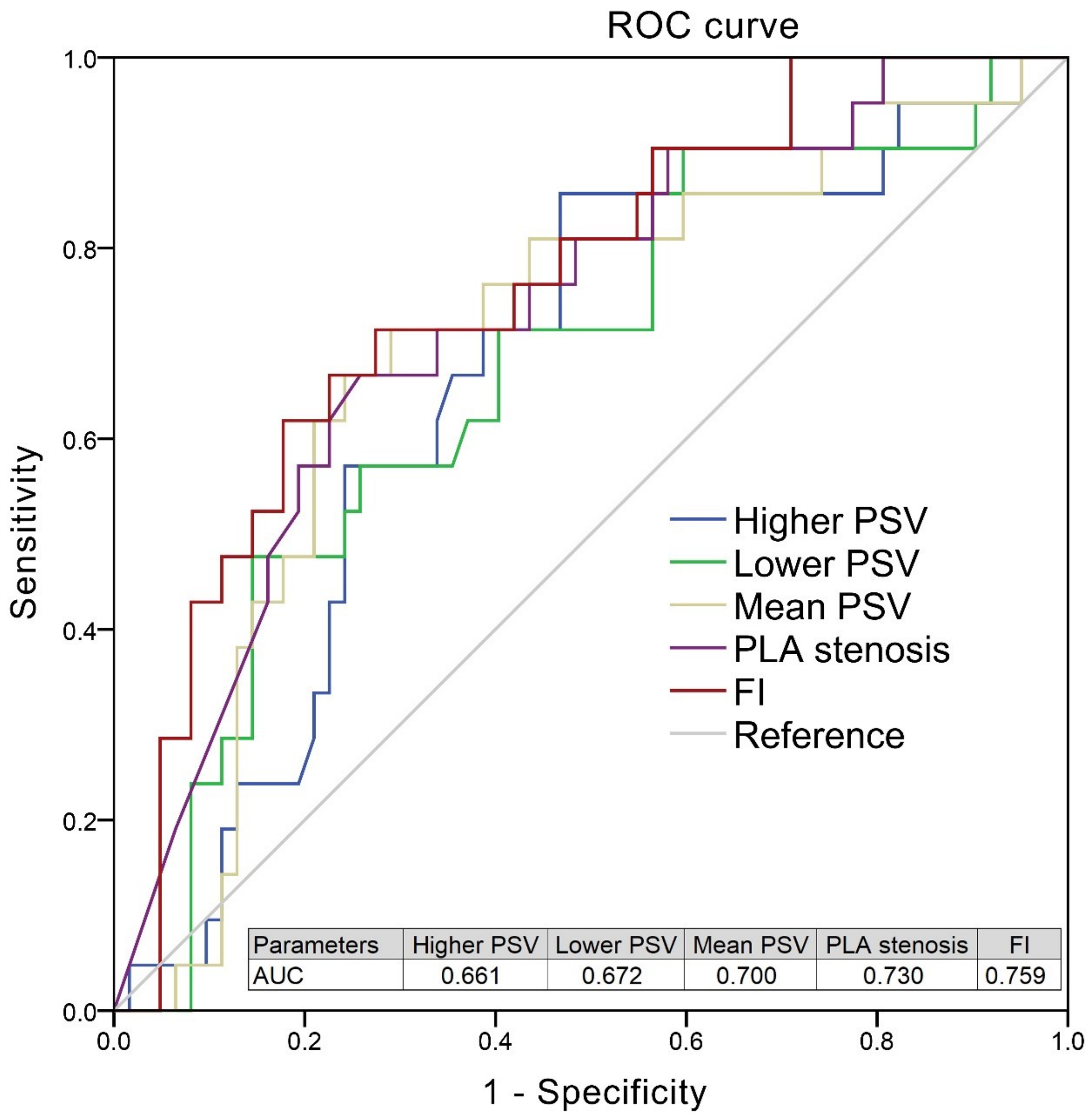

Parameters	Higher PSV	Lower PSV	Mean PSV	PLA stenosis	FI
AUC	0.661	0.672	0.700	0.730	0.759

P values of the DeLong test					
	Higher PSV	Lower PSV	Mean PSV	PLA stenosis	FI
Higher PSV	1.000	-	-	-	-
Lower PSV	0.863	1.000	-	-	-
Mean PSV	0.116	0.565	1.000	-	-
PLA stenosis	0.444	0.521	0.737	1.000	-
FI	0.166	0.246	0.394	0.334	1.000

Legends: The mean PSV, PLA stenosis, and FI exhibited acceptable AUCs in ROC analysis, while the higher and lower PSV exhibited fair AUCs; AUC = 0.5 (no discrimination), 0.5–0.6 (poor discrimination), 0.6–0.7 (fair discrimination), 0.7–0.8 (acceptable discrimination), 0.8–0.9 (excellent discrimination), and 0.9–1.0 (outstanding discrimination). The DeLong test of AUC revealed no significant difference between the AUCs. EHS: erection hardness score, FI: flow index, PSV: peak systolic velocity, PLA: pelvic artery.
